# Aluminosis – Detection of an almost forgotten disease with HRCT

**DOI:** 10.1186/1745-6673-1-4

**Published:** 2006-02-17

**Authors:** Thomas Kraus, Karl Heinz Schaller, Jürgen Angerer, Ralf-Dieter Hilgers, Stephan Letzel

**Affiliations:** 1 Institute and Outpatient-Clinic for Occupational and Social Medicine, Aachen University of Technology, Pauwelsstr. 30, D-52074 Aachen, Germany; 2 Institute and Outpatient-Clinic for Occupational, Social and Environmental Medicine of the University of Erlangen-Nuremberg, Schillerstr. 25 and 29, D-91054 Erlangen, Germany; 3 Institute for Medical Statistics, Aachen University of Technology, Pauwelsstr. 30, D-52074 Aachen, Germany; 4 Institute for Occupational, Social and Environmental Medicine of the University Mainz, Obere Zahlbacher Str. 67, 55131 Mainz, Germany

## Abstract

The aim of this study was to investigate whether it is possible to detect high-resolution computed tomography (HRCT) findings in aluminium powder workers, which are consistent with early stages of aluminosis.

62 male workers from 8 departments of two plants producing aluminium (Al) powder were investigated using a standardized questionnaire, physical examination, lung function analysis, biological monitoring of Al in plasma and urine, chest X-ray, HRCT and immunological tests.

Chronic bronchitis was observed in 15 (24.2%) of the workers, and four workers (6.5%) reported shortness of breath during exercise. HRCT findings in 15 workers (24.2%) were characterized by ill-defined centrilobular nodular opacities. Workers with ill-defined centrilobular nodular opacities had a lower vital capacity than workers who had no such HRCT-findings (90.9 % pred. vs. 101.8 % pred., p = 0.01). Biological monitoring in plasma and urine revealed higher internal exposure to Al in affected workers (33.5 μg/l plasma to 15.4 μg/l plasma, p = 0.01) and (340.5 μg/g creat. to 135.1 μg/g creat., p = 0.007). Years of exposure and concentration of aluminum in urine and plasma appear to be the best predictors for HRCT findings. Age and decreased vital capacity show borderline significance.

We conclude that aluminosis is still relevant in occupational medicine. With HRCT it is possible to detect early stages of aluminosis and biological monitoring can be used to define workers at high risk.

## Background

**Figure 1:**
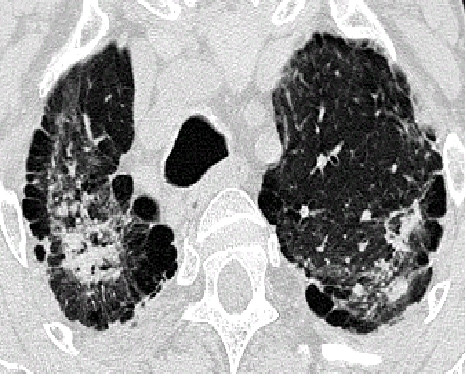
HRCT-scan of severe aluminosis with subpleural bullae.

The influence of the toxicity of aluminium and its compounds on humans has been the cause of much controversy for many years. Since the 1930's an 40's it has been known that high-level and long term occupational exposure to metallic aluminium powder and aluminium oxide can cause lung disease. At that time emphasis was placed on the short and long term effect of toxicity on the respiratory tract [4-7]. Recently the main discussion has been on the neurotoxicity and in particular on the controversial relationship between Alzheimer's disease and occupational or environmental exposure to aluminium [1-3]. It was assumed that under today's working conditions lung fibrosis induced by aluminium dust could not occur anymore [6,7]. However, several severe cases of aluminium-induced lung fibrosis have occurred in the last 15 years in Germany [8-10] (Fig. 1).

Histological examination of lung tissue samples showed severe subpleural and interstitial fibrosis with scar emphysema and spotted granulomateous pneumonitis with giant cells. Energy dispersed X-ray analysis of this case showed high concentrations of aluminium in the interstitial zones [9]. The pathogenesis of lung diseases induced by aluminium dust is still unclear. It has been much discussed whether only non-greased aluminium powder (pyro powder) or also greased aluminium powder can cause lung changes. The question is whether diverse additives, in particular stearic acids, are a pathogenetic factor in the development of lung fibrosis [4,5,8]. At first, diseases were detected in employees exposed only to high concentrations of stamped, non-greased aluminium flake powder. In the production process of aluminium powder, different amounts of stearic acid are added depending on its later use. Non-greased or barely-greased aluminium powder with a stearin content of less than 0.1 % (acetone extract) is used for sintered metals in powder metallurgy and in the production of fireworks, rockets and explosives in pyrotechnics. In other production fields (e.g. in the production of porous concrete and pigments for metallic paints) mainly greased aluminium powders are used in paste form or as granules with a lower exposure to dust. The threshold values for disease prevention, currently valid in Germany, are a maximum concentration at the workplace (MAK value) of 4 mg/m^3 ^as inhalable dust, 1.5 mg/m^3 ^as respirable dust and a biological tolerance value at the workplace (BAT value) of 200 μg/l in urine [11].

Aluminium lung is characterized as diffuse interstitial fibrosis which is mainly located in the upper and middle lobes of the lung. In advanced stages it is characterized by subpleural bullous emphysema with an increased risk of spontaneous pneumothorax [5]. The prognosis for severe forms of lung fibrosis is poor because the disease can continue to progress after the end of exposure. Therefore early detection of aluminium induced fibrotic changes is invaluable to the timely introduction of preventative measures. Early stage lung changes, induced by aluminium dust, could not be diagnosed to date using conventional X-rays in several cross-sectional studies in the aluminium powder industry [9] or during general occupational medical surveillance.

The aim of this study is to check whether sensitive tools for the detection of interstitial lung diseases, such as high resolution computed tomography (HR-CT), allow for the early detection of aluminium induced lung disease.

## Study group and Methods

### Study design

In a cross-sectional study, male workers were examined in two plants producing aluminium powder in Germany. The examination was offered to all workers from 8 departments, who had a high exposure to aluminium powder. In plant A, 34 of 76 high-exposed workers (44.7%) took part in the study. In plant B, 28 of 44 high-exposed workers (63.6%) from the production units gave their informed consent. None of the workers refused due to medical reasons to take part in the study. The age of the workers ranged between 22 and 64 years with a median of 41 years (mean 41.4, SD 9.9 yrs). The smoking history of the workers (20 non-smokers, 32 current smokers, 10 former smokers) was quantified by the cumulative cigarette consumption expressed in pack-years (PY).

The study design included a standardized history with special attention to occupational history including former exposures to fibrotic agents, a physical examination of the cardiopulmonary system, biological monitoring of aluminium in urine and in plasma, lung function analysis and conventional X-rays (first 28 consecutively examined workers only) and high resolution computed tomography with standardized technical parameters [12]. Sufficient data on ambient monitoring results from the two plants were not available retrospectively.

### Methods

Aluminium concentrations in plasma and urine were determined by graphite furnace atomic absorption spectrometry (GF-AAS) under the conditions of internal and external quality assurance [13,14]. Bodyplethysmography and spirometry were performed with a Jaeger-Masterlab (Jaeger-Toennies GmbH, Germany) according to ATS criteria [15]. It included measurements of the vital capacity (VC), forced expiratory volume (FEV1), total resistance (Rtot), and total lung capacity (TLC). Lung function measurements relative to the corresponding reference values proposed by the European Community for Coal and Steel [16] were used in the analysis. Evaluation of the conventional X-rays was performed using the ILO-classification for pneumoconiosis [17], by an experienced blinded (no knowledge of the quantitative exposure or clinical data) radiologist. After the first 28 consecutive chest X-ray examinations, this method was discontinued due to the lack of aluminium-related findings in the chest X-rays. The HRCT was performed during breath-holding at full inspiration with a Somatom plus4 scanner from Siemens, Erlangen. Slice thickness was 1 mm with a slice interval of 10 mm. The evaluation of the CT scans was performed with a semi-quantitative score system for CT [12]. Similar to the ILO-Classification for pneumoconioses small rounded opacities, irregular and linear opacities, emphysema, honeycombing and ground glass pattern as well as pleural plaques and diffuse pleura thickening were quantified as profusion grade (parenchyma) and thickness and extent (pleura).

In the case of suspected aluminium-related findings, further diagnostic tests were performed to exclude other interstitial lung diseases. These tests included ergometry, diffusion capacity (DLCO single breath method), blood gas analysis, and immunological parameters (Table 5). These parameters were C-reactive protein, anti-ribonuclease, rheumatoid factor, Rose-Waaler test, antinucleic antibodies (ANA) fluorescence test, ribonucleoprotein /Sm antibodies, U1-ribonucleoprotein antibodies, sm antibodies, Sjoegren-syndrome-A-antibodies (Ro and La), sclerodermia-70-antibodies, CENP-B-antibodies, anti-Jo-antibodies, antimitochondrial antibodies and neutrophile cytoplasmatic antibodies (AK/C and AK/P). Specific IgG antibodies were analyzed for Penicillium notatum, Cladosporium herbarum and Aspergillus fumigatus. Specific IgE antibodies were analyzed for grasses, tree pollen (beech, alder, birch, hazel), flakes of cat skin, mold (Penicillium notatum, Cladosporium herbarum, Aspergillus fumigatus), household dust and dust mites.

Informed written consent was obtained from each participant. The protocol was approved by the Ethics Committee of the Medical School of the University Erlangen-Nuremberg, Germany.

### Statistical analysis

The data were described by means, standard deviations and proportions.

We used Pearson correlation to investigate the correlation between aluminium concentrations in plasma and urine.

Unpaired t-test were used to find univariate distributional differencies between the cases (occupational disease = yes) and non cases (occupational disease = no) with respect to age (years), weight (kg), height (cm), time of exposure (months), Al -plasma (μg/l), Al -urine (μg/gcreat), FEV1/VC (%), TLC (% pred.), VC (% pred.), Rtot (kPa*s/l) and body mass index. Moreover differencies between cases and non-cases in the distribution of smoking habits was analysed using χ^2 ^test. The next step of the analysis is addressed to the question of the multivariate dependency between several independent factors and the occurrence of an occupational disease (aluminosis). If the univariate p-value of distributional differencies was below 0.40 the corresponding independent factors was included in the multivariate model. The margin p ≤ 0.4 is chosen to be rather conservative, because of the limited sample size. Thus the multivariate associations between the occurrence of an aluminosis and age, sex, smoking habits, lung function parameters (vital capacity, total resistance, forced expiratory volume) and biological monitoring were studied using a logistic regression model. Differences with a p-value smaller or equal to 0.05 were regarded as significant.

Statistical analysis were performed using proc ttest, freq and logist with SAS^® ^software.

## Results

### Occupational and disease history

The 62 male workers (plant A: 28; plant B: 34) were exposed to aluminium powder for a median of 123 months (range 13 – 360 months) as stampers (n = 11), polishers (n = 7), dryers (n = 6), packers (n = 4), mixers (n = 10), ball mill operators (n = 11) and others as controllers, metalworkers etc. (n = 7). Former exposure to fibrotic agents was reported by 14 workers. 11 were exposed to asbestos as construction workers (n = 3), metalworkers (n = 6) and car mechanics (n = 2), and 3 to silica dusts. Exposure to other fibrotic agents at the current workplace (e.g. other metals including cobalt, beryllium etc.) can be excluded. 15 workers reported a chronic cough and phlegm, 11 of them were smokers. 9 had a positive history of pneumonia, pleuritis or tuberculosis. Four workers reported shortness of breath during exercise.

### Biological monitoring

**Figure 2:**
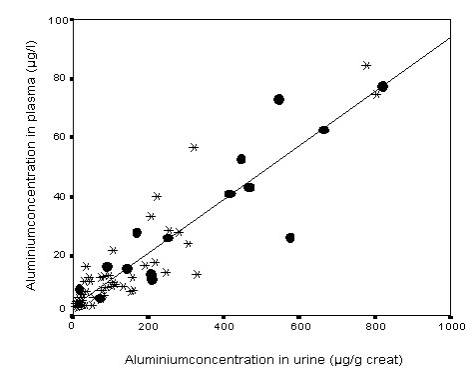
Correlation of Al concentrations in plasma and urine (marked dots are workers with early aluminosis).

The median aluminium concentration in plasma was 12.5 μg/l (range 2.5 – 84.4 μg/l) and in urine 83.3 μg/l (range 3.7 – 630.0 μg/l) or 104.3 μg/g creat. (range 7.9 – 821.2 μg/g creat.). The BAT value of 200 μg/l urine was exceeded in 20 cases (32.3 %). The aluminium concentrations in plasma and urine showed a significant correlation (r = 0.83) related to the urinary Al concentration in μg/l and r = 0.93 related to μg/gcreat. (Figure 2).

The intensity of exposure depended on the workplace area. A detailed description of the internal aluminium exposure at the different workplaces is shown in Table 1. The highest aluminium concentrations in biological materials were found in stampers.

**Table 1:** Mean, median and standard deviation (SD) of aluminium concentrations in plasma and urine at different workplaces

			**Al-plasma (μg/l)**			**Al-urine (μg/g creat)**		
	**No.**	**%**	**mean**	**median**	**S.D.**	**mean**	**median**	**S.D.**

polisher	7	11.3	16.3	5.7	21.5	189.5	76.6	232.3
dryer	6	9.7	8.4	8.9	3.8	60.9	62.8	40.2
stamper	11	17.7	40.7	40.1	25.9	382.2	415.7	273.1
packing	4	6.5	14.0	12.0	10.8	192.2	193.7	124.2
mixing	10	16.1	23.1	12.3	24.3	195.8	132.9	215.2
ball mills	11	17.7	9.9	7.4	5.8	80.2	27.0	86.5
sieving	6	9.7	21.9	15.0	18.3	227.7	175.1	198.4
others (controller, metalworker)	7	11.3	12.1	12.8	10.0	83.4	44.7	92.9

### Chest X-rays

Chest X-rays were performed with the first 28 workers investigated. In 3 patients small rounded and irregular opacities with a profusion of 1/0 (n = 2) and 1/2 (n = 1) according to the ILO-classification were found. The findings were described by the radiologist as non-specific.

### HRCT-findings

HRCT revealed in 15 of 62 workers (24.2%) parenchymal changes of the same pattern. This was characterized by small rounded opacities predominantly in the upper lung regions. Moreover there were signs of a beginning thickening of the interlobular septae in three cases. In four cases these opacities were located additionally in the middle and lower lobes. The rounded opacities had a maximum diameter of 3 mm. 9 of the 15 workers with rounded opacities had worked as stampers and were exposed to barely greased or non-greased aluminium-flake powder.

10 of 15 workers with HRCT findings were found to have aluminium concentrations in urine above the threshold limit value of 200 μg/l (Fig. 2).

**Figure 3:**
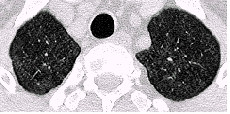
HRCT-scans. In the whole lung area there are small, ill-defined, diffuse opacities, in the upper right-hand field subpleural curvilinear lines. Figure 3 upper field, figure 4 middle field, Figures 5 and 6 lower field (case 10, table 5 and 6) [18]

**Figure 4:**
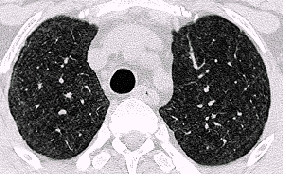
HRCT-scans. In the whole lung area there are small, ill-defined, diffuse opacities, in the upper right-hand field subpleural curvilinear lines. Figure 3 upper field, figure 4 middle field, Figures 5 and 6 lower field (case 10, table 5 and 6) [18]

**Figure 5:**
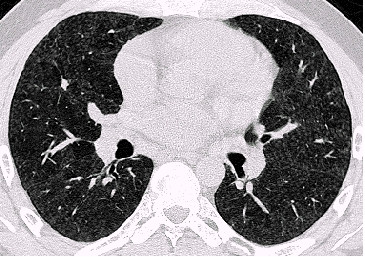
HRCT-scans. In the whole lung area there are small, ill-defined, diffuse opacities, in the upper right-hand field subpleural curvilinear lines. Figure 3 upper field, figure 4 middle field, Figures 5 and 6 lower field (case 10, table 5 and 6) [18]

**Figure 6:**
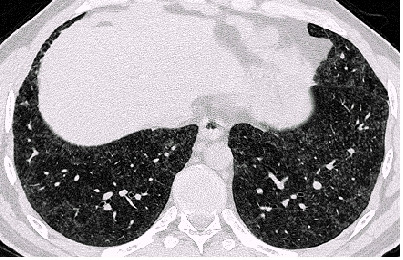
HRCT-scans. In the whole lung area there are small, ill-defined, diffuse opacities, in the upper right-hand field subpleural curvilinear lines. Figure 3 upper field, figure 4 middle field, Figures 5 and 6 lower field (case 10, table 5 and 6) [18]

Examples of the parenchymal changes are shown in Figures 3, 4, 5 and 6. This example has been published as a case report with detailed informations on the diagnostic procedures and results [18].

### Workers with aluminium-induced CT-findings

Workers with HRCT changes had worked as stampers (n = 9), polishers (n = 2), ball mill operators (n = 2), mixers (n = 1) and sievers (n = 1). Affected workers had higher concentrations of aluminium in plasma (AI-plasma, p = 0.01) and urine (AI-urine, p = 0.003) and a lower vital capacity (p = 0.01) (table 3). The age, time of exposure, total lung capacity (TLC), resistance (Rtot), and the results of the Tiffeneau-test (FEV1/VC) did not differ between workers with and without lung changes induced by aluminium dust in the univariate comparison between the groups (table 3). Smoking habits, including number of pack-years, had no influence on the prevalence of HRCT changes (χ^2 ^test, p = 0.5028) (table 2). Parenchymal changes did not correlate with the existance of respiratory symptoms. Higher (200 and more) aluminium concentrations in urine (with relation to creatinine) and higher (120 days and more) duration of exposure were significantly associated with aluminosis. Vital capacity and FEV_1_/VC were factors of borderline significance (table 4). Including aluminium concentration in urine without correction for creatinine and aluminium concentration in plasma into the regression model yielded to similar results. With these variations the model fit was slightly worse.

**Table 2:** Smoking habits in workers with and without aluminium-induced findings (% in brackets)

	**Smoking habits**			
	**Non-smokers**	**Current smokers**	**Former smokers**	**total**

no aluminosis	17 (27.4)	23 (37.1)	7 (11.3)	47 (75.8)
aluminosis	3 (4.8)	9 (14.5)	3 (4.8)	15 (24.2)
Total	20 (32.6)	32 (51.6)	10 (16.1)	62 (100)

**Table 3:** Anamnestic, lung function data and biological monitoring in workers with and without HRCT findings (t-test)

Parameter	Al-induced findings				
	
	no		yes		
	
	Mean	Std.Err.	Mean	Std.Err.	Pr>|t|
Age (years)	40.9	1.6	42.9	1.8	0. 3855
Weight (kg)	82.9	2.0	82.0	3.7	0. 8221
Height (cm)	172.9	1.1	173.5	1.7	0. 7726
Time of exposure (months)	142.7	15.2	180.0	21.7	0.1700
Al -plasma (μg/l)	15.4	2.5	33.5	6.1	0.0124
Al -urine (μg/gcreat)	135.1	24.5	340.5	62.4	0. 0065
FEV1/VC (%)	83.9	0.8	86.0	0.7	0.0660
TLC (% pred.)	104.6	1.9	95.4	5.1	0.1070
VC (% pred.)	101.8	2.1	90.9	3.5	0.0133
Rtot (kPa*s/l)	0.2	0.0	0.2	0.0	0.1363
Body mass index	27.8	0.6	27.3	1.1	0.6951

**Table 4:** Logistic regression analysis of factors predicting the occurrence of HRCT changes

Analysis of Maximum Likelihood Estimates						Odds Ratio Estimates		
Parameter	DF	Estimate	Standard Error	Wald Chi-Square	Pr>ChiSq	Point Estimate	95% Wald Confidence Limits	

Intercept	1	-14.5072	10.9830	1.7447	0.1865			
Age	1	-0.0571	0.0630	0.8217	0.3647	0.944	0.835	1.069
Time of exposure	1	0.0152	0.00681	4.9905	0.0255	1.015	1.002	1.029
Smoking habits	1	0.4164	1.1040	0.1423	0.7060	1.516	0.174	13.200
Vital capacity	1	-0.0664	0.0371	3.2085	0.0733	0.936	0.870	1.006
FEV1/VC	1	0.2206	0.1231	3.2149	0.0730	1.247	0.980	1.587
Resistance	1	-4.9536	5.8181	0.7249	0.3945	0.007	<0.001	632.518
Al (urine(creat.)	1	0.00768	0.00278	7.6541	0.0057	1.008	1.002	1.013

**Table 5:** Anamnestic data and biological monitoring results in 15 affected workers with HRCT findings

Case No.	age	workplace	duration of exposure (months)	Al-plasma (μg/l)	Al-urine (μg/gcreat)	smoking habits/packyears		other fibrotic agents	cough	phlegm	shortness of breath
1	39	polisher	122	62.3	665.6	current	9.60	asbestos	-	-	+
2	35	stamper	113	42.9	467.8	former	8.00	asbestos	+	-	-
3	39	stamper	150	28.1	172.6	current	24.00	silica	-	-	-
4	42	ball mill	120	16.6	91.1	current	13.20	asbestos	+	+	-
5	48	stamper	78	73.0	545.9	non	-	no	-	-	-
6	41	stamper	124	52.8	446.8	non	-	no	-	-	-
7	31	mixing	156	14.0	207.4	non	-	no	-	-	-
8	48	polisher	312	5.7	72.2	current	22.00	no	+	+	+
9	50	stamper	360	8.9	17.3	current	51.00	no	+	+	+
10	39	stamper	174	41.0	415.7	former	3.30	no	+	+	+
11	53	stamper	258	12.2	210.8	current	11.40	no	+	+	-
12	55	stamper	198	77.0	821.2	current	29.25	no	-	-	-
13	35	ball mill	276	16.0	142.3	current	29.00	no	-	-	-
14	45	sieving	96	26.4	577.5	current	15.50	no	-	-	-
15	44	stamper	163	256.0	253.0	former	16.00	no	-	-	-

4 of 15 affected workers (26.7%) and 10 of 42 (23.8%) non-affected workers were exposed to fibrotic agents in former occupations. 5 affected workers reported symptoms of chronic bronchitis, 4 reported shortness of breath induced by exercise. During further medical work-up of the 15 affected workers, exercise induced decrease in pO_2 _occured in 4 cases (table 5, Nos. 2,10,12,14). 8 patients presented positive results in immunological tests for specific IgE, indicating sensitization to environmental antigens. None of them had any symptoms which suggested a clinical relevance of these findings. Auto-antibodies were slightly positive in three cases (n = 2 ANA, ANA normal value < 1:10; sjoegren syndrome antigen La normal value < 1) without clinical signs of a corresponding disease (table 6). In 11 of 15 cases results from biological monitoring of Al in plasma were available from former years. The Al-concentrations ranged between 9.8 μg/l and 183 μg/l (median 85 μg/l, arithmetic mean 84.6 μg/l) (table 6).

**Table 6:** Lung function data and results of the immunological tests in 15 affected workers with HRCT findings.

Case No.	VC %pred	FEV1/VC (%)	TLC %pred.	R_tot (kPa*l/s)_	Diff.Cap. (%)	pO_2_	pCO_2_	spec. IgE	spec. IgG	Autoanti- bodies	Maximum Al-conc. in plasma (μg/l) since 1980
1	83.0	85.4	65.10	0.24	119	→	→	neg	neg	neg	62.3
2	82.8	80.7	98.70	0.14	89	↓	→	pos^*5^	neg	neg	112.7
3	99.8	82.9	82.80	0.30	116	↑	→	pos^*6^	neg	neg	29.5
4	96.4	89.2	69.00	0.27	105	↑	→	pos^*1^	neg	ANA pos 1:20	9.8
5	87.0	88.6	66.90	0.24	99	↑	→	pos^*7^	neg	neg	106.2
6	95.3	86.1	97.80	0.03	95	→	→	neg	neg	neg	85.0
7	84.3	86.5	139.00	0.12	97	↑	→	neg	neg	neg	28.1
8	100.0	90.9	85.70	0.15	88	↑	→	pos^*2^	neg	ANA pos.1:20	-
9	83.6	86.6	121.00	0.31	64	↑	→	neg	neg	neg	-
10	57.5	88.6	68.20	0.12	71	↓	→	pos^*4^	neg	neg	170.1
11	105.0	83.9	142.00	0.09	117	↑	→	pos^*3^	neg	neg	45.0
12	86.7	84.5	72.70	0.39	102	↓	→	neg	neg	neg	183.0
13	120.0	88.3	125.00	0.14	-	↑	→	pos^*8^	neg	neg	-
14	90.8	83.4	111.00	0.21	100	↓	↑	neg	neg	SS-B-AG La 1.9	-
15	91.2	83.9	99.10	0.23	110	↑	→	neg	neg	neg	100.5

Antigens Class

^*1 ^Ragweed, birch, dust mites 2

Alder, hazel 1

^*2 ^Penicillium notatum, dust mites 3

Houshold dust, Aspergillus fumigatus 2

Ragweed, Cladosporium herbarum 1

^*3 ^Asp. fumigatus 1

^*4 ^Dust mites 2

^*5 ^Dust mites 1

^*6 ^Grasses 2

Dust mites, flakes of cat skin, household dust 3

^*7 ^Penicillium notatum 3

^*8 ^Dust mites 3

## Discussion

Lung diseases induced by aluminium dust are very rare in occupational medicine. Between 1960 and 1989 only a few individual cases were identified, mainly in the aluminium powder industry. It was assumed that under today's working conditions lung fibrosis induced by aluminium dust was virtually non-existant [6,7]. In former times, it was even proposed that workers exposed to silica inhale aluminium lactate to suppress the development of silicosis [19,20]. Since the beginning of the 90s, however, several cases of severe fibrosis have been recognized by the employers'liability insurance and financially compensated in Germany [8]. Young men with only short periods of exposure were also affected and the prognosis was poor [10]. In other aluminium industries the existence of aluminium-induced lung diseases is the subject of much controversy [21,22]. In most studies, especially in cross-sectional studies of workers exposed to aluminium, no increase in the prevalence of pneumoconiotic changes was found using conventional chest X-rays [9,23]. In one study, Townsend et al [24] classified an increase in small irregular opacities in aluminium smelters as non-specific changes. De Vuyst et al [25] reported severe lung fibrosis in an aluminium polisher. Early stages of aluminosis have not yet been described.

In recent years the use of high resolution computed tomography (HRCT) has proved very reliable for the detection of occupationally induced pneumoconiosis [26]. In several studies HRCT could be shown to have higher sensitivity and specificity compared to conventional chest X-rays, in particular for asbestos-related diseases [26-28]. So far there are only case reports available on the use of HRCT with workers exposed to aluminium dust [10,29]. They described advanced stages of aluminosis. The predominant CT findings consist of subpleural bullae, and parenchymal changes with distortion of intrathoracal structures. Early stages of aluminosis have not been specified using either conventional X-rays or CT.

In 15 of 62 high exposed workers we were able to detect early stages of aluminosis for the first time using HRCT. The CT findings are specified by small rounded and ill-defined centrilobular opacities mainly in the upper lobes which cannot be assessed using chest X-rays. The CT findings suggest beginning alveolitis, without fibrotic activity. Severe cases from the same plants show that there is a considerable risk of these early stages progressing to severe fibrosis [9] (Fig. 1). Unfortunately the 15 affected workers in this study refused to undergo bronchoscopy so that no biopsy results are available. Immediate intervention took place to reduce aluminium exposure in both plants. Affected workers were removed from workplaces with high exposures.

Fig 2 shows that not all highly exposed workers were found to have parenchymal changes. This suggests that individual susceptibility plays an important role in the development of aluminosis. Neither the smoking habits nor cumulative cigarette consumption in pack-years differ between affected and non-affected workers (Tables 2 and 3). As stampers and subjects with increased and longer exposure were over-represented in the affected group, type, duration and intensity of exposure seem to be the most important risk factors besides unknown individual ones. Stampers are exposed to a very fine flake powder with a high proportion of flakes with a diameter below 5 μm. Lung function analysis has a low sensitivity for detecting affected workers and is therefore not an appropriate tool for screening exposed workers. Affected workers, however, had a 10 % lower vital capacity than non-affected workers on a group basis (Table 3).

All workers have had regular medical check-ups involving anamnesis, lung function tests and chest X-rays not exhibiting early stages of aluminosis. When interpreting the significant correlations between Al-concentrations in plasma and urine and the presence of aluminosis, it has to be considered that the results of biological monitoring represent acute exposure while the development of aluminosis is likely to be a chronic effect. In 11 of 15 affected workers, results from biological monitoring of Al in plasma were available (table 5b). These show that Al exposure has been, at least during the last 10 years, very high. For diagnostic purposes HRCT proved to be more sensitive and specific than chest X-rays for identifying lung disease induced by aluminium dust. However, it is not possible to use HRCT as a screening tool in an undifferentiated way because of the high costs and considerably higher radiation exposure compared to chest X-rays. For the selective use of HRCT, high-risk groups must be defined on the basis of risk factors [26]. Our study showed that job classification, e.g. working as a stamper for many years and, high aluminium concentrations in plasma and urine are the best markers of workers at risk.

## Pathogenetic considerations

Radiomorphological patterns suggest that aluminosis develops from alveolitis, as has been shown for other pneumoconiotic diseases [31]. Long-term follow-up of the affected workers will show whether and to what extent regression of the disease is possible. Etiologic agents and pathogenetic considerations other than aluminium cannot be supported.

Arguments for aluminium-induced changes are supported by (1) the consistent pattern in all affected workers, (2) the fact that there is a dose-dependency in the findings (3) that the changes were found in two different plants and (4) the lack of results that would support another hypothesis.

The exposure of 3 workers to asbestos and of 1 worker to crystalline silica cannot be responsible for the radiological findings in those cases. The sensitization of 8 affected workers to environmental antigens is without clinical relevance because none of them reported characteristic symptoms. Moreover, type-I sensitization does not lead to alveolitic changes in the lungs. Specific IgG antibodies or symptoms that are typical of hypersensitivity pneumonitis due to environmental antigens could not be found. The three slightly positive antibodies (two ANA, one SS-AG-La) are without clinical relevance because there were no other findings suggesting an auto-immune disease of any kind.

In the scientific literature it has been discussed for many years whether only non-greased aluminium powder or special additives such as stearic acid are responsible for the development of fibrosis induced by aluminium dust [4,5,8]. In our group all participants were exposed to a mixture of non-greased and at least barely greased aluminium powder. Parenchymal changes induced by aluminium dust were present mainly in workers that were exposed to barely or non-greased aluminium powder at the stamping workplaces. The highest exposures to aluminium dust exist at these workplaces and most of this aluminium dust is respirable with a diameter smaller than 5 μm. Lung changes induced by aluminium dust in workers that were exposed to only greased aluminium powder could not be detected in our study. Barely greased or non-greased aluminium powder is therefore thought to be the main pathogenetic risk factor for the development of lung fibrosis induced by aluminium dust, although it is still not clear whether greased aluminium powder alone can cause aluminium-induced lung diseases.

## Conclusion

Aluminium is of growing importance in industry and adequate substitutes will not be available in the near future. Our findings show that aluminosis is still relevant in occupational medicine. Probably the detection of early stages of aluminosis is not due to a recurrence of a historical disease but to the use of more sensitive diagnostic tools. However, it is important that in addition to a reduction in exposure also specific and efficient measures of secondary prevention are implemented. Biological monitoring is the most easily available and suitable tool for the identification and screening of high risk groups [30]. Our findings also show that in high-risk groups, HRCT can be an important complementary tool for the early detection of aluminosis.
